# Effects of Adult Aging on Letter Position Coding in Reading: Evidence From Eye Movements

**DOI:** 10.1037/pag0000342

**Published:** 2019-03-28

**Authors:** Kayleigh L. Warrington, Victoria A. McGowan, Kevin B. Paterson, Sarah J. White

**Affiliations:** 1 Department of Neuroscience, Psychology and Behaviour, University of Leicester

**Keywords:** reading, eye movements, aging, letter position coding, transposed letters

## Abstract

It is well-established that young adults encode letter position flexibly during natural reading. However, given the visual changes that occur with normal aging, it is important to establish whether letter position coding is equivalent across adulthood. In 2 experiments, young (18–25 years) and older (65+ years) adults’ were recorded while reading sentences with words containing transposed adjacent letters. Transpositions occurred at beginning (*rpoblem*), internal (*porblem*), or end (*problme*) locations in words. In Experiment 1, these transpositions were present throughout reading. By comparison, Experiment 2 used a gaze-contingent paradigm such that once the reader’s gaze moved past a word containing a transposition, this word was shown correctly and did not subsequently change. Both age groups showed normal levels of comprehension for text including words with transposed letters. The pattern of letter transposition effects on eye movements was similar for the young and older adults, with greater increases in reading times when external relative to internal letters were transposed. In Experiment 1, however, effects of word beginning transpositions during rereading were larger for the older adults. In Experiment 2 there were no interactions, confirming that letter position coding is similar for both age groups at least during first-pass processing of words. These findings show that flexibility in letter position encoding during the initial processing of words is preserved across adulthood, although the interaction effect in rereading in Experiment 1 also suggests that older readers may use more stringent postlexical verification processes, for which the accuracy of word beginning letters is especially important.

Compared to young adults (aged 18–30 years), healthy older adults (aged 65+) generally have longer sentence reading times, despite achieving normal levels of comprehension (Kliegl, Grabner, Rolfs, & Engbert, 2004; McGowan, White, Jordan, & Paterson, 2014; McGowan, White, & Paterson, 2015; Paterson, McGowan, & Jordan, 2013a, 2013b; Rayner, Reichle, Stroud, Williams, & Pollatsek, 2006; Stine-Morrow et al., 2010; Warrington, McGowan, Paterson, & White, 2018; Warrington, White, & Paterson, 2018). Being able to read well is essential to function effectively in modern societies, and so it is important to understand whether mechanisms that underlie successful reading change in older age. One possibility is that older adult readers differ in their processing of words compared to young adult readers as a consequence of age-related visual declines. Older adults show reduced sensitivity to fine visual detail (Crassini, Brown, & Bowman, 1988; for a review, see Owsley, 2011), which may make recognition of individual letters more challenging. Older adults are also more sensitive to the effects of visual crowding (i.e., the impaired ability to identify an object when it is flanked by similar objects, for a full definition, see Bouma, 1970; for evidence of greater crowding effects in older age, see Liu, Patel, & Kwon, 2017; Scialfa, Cordazzo, Bubric, & Lyon, 2013). This increased crowding may be detrimental for coding the positions of adjacent letters. Visual changes in older age may therefore affect the processing of letters at different positions in words.

In addition, other evidence suggests age differences in how words are processed that may affect the use of letter position information. For instance, there is evidence that older adults process words more holistically, and so may rely less on processing of the component features of a word, such as its individual letters (Spieler & Balota, 2000). There is also evidence that older adults’ greater cumulative experience with lexical processing allows word recognition to become more automatized (Lien, Allen, Ruthruff, Grabbe, McCann, & Remington, 2006). The present study therefore aims to reveal whether letter position coding is modulated by adult age.

The encoding of letter positions is an important process contributing to word recognition. Accurate encoding of letter position allows readers to distinguish anagrams, such as *calm* and *clam.* Further, individual letters are thought to be the unit that provides information input to more complex subsequent sublexical and lexical processes (Schoonbaert & Grainger, 2004). Given the importance of individual letters and their order, a considerable amount of research has focused on how letter position is encoded (e.g., Andrews, 1996; Forster, Davis, Schoknecht, & Carter, 1987; Grainger & Whitney, 2004; Perea & Fraga, 2006; Perea & Lupker, 2003a, 2003b, 2004; Perea, Rosa, & Gómez, 2005; Rawlinson, 1999; Schoonbaert & Grainger, 2004; for a recent review, see Grainger, Dufau, & Ziegler, 2016). For young adult readers of European languages such as English, there is now a wealth of evidence demonstrating that letter position coding is flexible rather than fixed (for a review of letter position coding in other languages, see Frost, 2015).

Letter position coding has been studied experimentally using several paradigms. A common approach involves using pseudowords created by swapping two (usually adjacent) letters within a word (e.g., presenting *ujdge* in place of *judge*), known as transposed letter (TL) nonwords. Studying the processing of these nonwords can help reveal how individual letters and their positions are encoded, and so provide insights into the nature of word recognition processes. Single word priming tasks have demonstrated that TL nonwords are more effective as a prime in lexical decision tasks than words formed using letter substitutions (Perea & Lupker, 2003a, 2003b, 2004). In addition, TL nonwords produce associative priming (e.g., *jugde* primes *COURT*; Perea, Palti, & Gómez, 2012) and provide as much facilitative priming as correctly spelled primes in naming tasks (Christianson, Johnson, & Rayner, 2005). In line with this work, sophisticated models of word recognition have been developed that incorporate flexible letter position coding (e.g., Davis & Bowers, 2006; Gómez, Ratcliff, & Perea, 2008; Grainger & Van Heuven, 2003; Whitney, 2001).

The present study focuses on letter position coding during natural reading, by examining the reading of sentences in which some words have transposed letters. For young adults reading English, several studies show, in line with the notion of flexible letter position coding, that comprehension is good for sentences containing TL nonwords, although letter transpositions are associated with longer reading times (Blythe, Johnson, Liversedge, & Rayner, 2014; Johnson & Eisler, 2012; Rayner, White, Johnson, & Liversedge, 2006; White, Johnson, Liversedge, & Rayner, 2008; see also: Johnson, 2009; Johnson & Dunne, 2012; Velan & Frost, 2007). Such studies demonstrate flexibility in letter position encoding during natural reading, and that words containing letter transpositions are recognized rapidly and sentences containing these words readily understood. Overall, there is now strong evidence suggesting that TL nonwords enable rapid access to the lexical representation of their base word. A crucial question concerns whether these effects are stable across different age groups. Most studies have investigated effects in skilled young adult readers, whereas a growing number of studies have begun to investigate the development of letter position encoding during childhood (Grainger, Bertrand, Lété, Beyersmann, & Ziegler, 2016; Grainger, Lété, Bertand, Dufau, & Ziegler, 2012; Kezilas, McKague, Kohnen, Badcock, & Castles, 2017; Marinus, Kezilas, Kohnen, Robidoux, & Castles, 2018; Perea & Estévez, 2008; Perea, Jiménez, & Gomez, 2016; see also Paterson, Read, McGowan, & Jordan, 2015). However, studies to date have not examined adult ageing effects, and so it will be important to determine if older adults also use flexible letter position encoding to recognize words during reading.

Importantly, studies with young adults have shown that not all letter positions contribute equally to the process of word recognition. Numerous studies reveal a privileged role for the external letters in words (Carr, Lehmkuhle, Kottas, Astor-Stetson, & Arnold, 1976; Forster, 1976; Guérard, Saint-Aubin, Poirier, & Demetriou, 2012; Johnson & Eisler, 2012; Jordan, Thomas, Patching, & Scott-Brown, 2003; Rayner & Kaiser, 1975) and especially the first letter (Aschenbrenner, Balota, Weigand, Scaltritti, & Besner, 2017; but see Winskel, Ratitamkul, & Perea, 2018). Similarly, studies of transposed letter effects on sentence reading (Johnson & Eisler, 2012; Johnson, Perea, & Rayner, 2007; Rayner, White, et al., 2006; White et al., 2008) show that normal reading is disrupted more when transpositions are made at exterior rather than interior letter locations in words, with transpositions of the beginning letters most disruptive of all. One explanation why external letters are particularly important is that they are easier to encode because they are less crowded (e.g., Grainger, Tydgat, & Isselé, 2010; Levi, 2008; Pelli, Tillman, Freeman, Su, Berger, & Majaj, 2007). That is, interior letters are most crowded because they are flanked on both sides, whereas exterior letters are flanked on only one side and so there is less crowding when spaces are included between words in sentences. Note that as older adults are more sensitive to the effects of crowding than young adults (Liu et al., 2017; Scialfa et al., 2013), such effects may be even greater for older readers and so the external letters of words may be especially important to their word recognition.

The greater importance of beginning letters, compared to end letters, suggests that initial and final letters may contribute to word recognition through different processes. In line with this, Johnson and Eisler (2012) found that when crowding for letters was equated (by filling the spaces between words with #), the first letter of a word retained its privileged role over interior letters. However, the word final letter no longer had a privileged role. Johnson and Eisler concluded that the importance of word ending letters arises from low-level visual factors, whereas word beginning letters have intrinsic importance for word recognition (Davis & Bowers, 2006; Gómez et al., 2008; Whitney, 2001). Various explanations for this have been proposed, including that letters within a word are processed serially in left-to-right order (e.g., Kwantes & Mewhort, 1999; see Adelman, Marquis, & Sabatos-DeVito, 2010, for evidence against this notion), although this does not account for the more important role of word ending letters relative to interior letters (see also model descriptions below). Alternatively, beginning letters may be important in constraining the number of lexical candidates for a word (Broerse & Zwaan, 1966; Clark & O’Regan, 1999; Hand, O’Donnell, & Sereno, 2012; Lima & Inhoff, 1985).

Models of word encoding also differ in their predictions about the role of letters in different positions. The SOLAR model (Self-Organizing Lexical Acquisition and Recognition; Davis, 1999) relies on spatial coding whereby letter nodes are activated by all constituent letters, with activation reducing as a function of left to right position within a word. The model may therefore predict that internal letters are more important than end letters, and so transposing internal letters within a word should be more disruptive than transposing end letters (which does not fit with the observed findings, see Johnson & Eisler, 2012). Though note that more recent extensions to this model have sought to accommodate the possibility that external letters are assigned greater weight when performing similarity calculations, see Davis and Bowers (2006). By comparison, the SERIOL model (Sequential Encoding Regulated by Inputs to Oscillations within Letter units; Whitney, 2001) uses continuous open bigrams which encode constituent letters as all bigrams that can be formed from the word (e.g., dog would be encoded as DO, OG and DG). However, the bigrams are weighted for adjacency so that bigrams have higher activation if the component letters are closer together. The model also specifies that crowding from adjacent letters can reduce activation. Thus, external letters are advantaged over internal letters, and the model predicts that transposition of external letters is more disruptive than transposition of internal letters. All of these models focus on optimized processes associated with skilled reading performance and none make explicit predictions regarding changes in letter position coding that may occur across the life span. However, an approach based on continuous open bigrams (e.g., the SERIOL model, Whitney, 2001) may predict greater disruption for older adults when external letters are transposed, as increased visual crowding for internal letters may result in greater reliance on external letters.

To summarize, studies with young adults have demonstrated flexibility in letter position coding and also that external letters, and especially word-initial letters, have particular importance for normal, efficient processing. Most research to date has focused on young adults. However, visual declines in older age (Crassini et al., 1988; Scialfa et al., 2013; see Owsley, 2011) and potential aging effects on the processing of words (Lien et al., 2006; Spieler & Balota, 2000) may produce adult age differences in the flexibility of letter position coding, or the relative importance of letters at different positions within words (beginning, internal, ending) during reading.

## Experiment 1

Experiment 1 aimed to (a) establish whether young and older adults’ eye movements differ in response to words with transposed letters and (b) examine whether this pattern holds for transpositions in different letter positions. To achieve this, young and older adults read sentences including words with transposed adjacent letters. In addition to a normal control condition (no transpositions), the transposition types used were word beginning transpositions (*rpoblem*), internal transpositions (*porblem*), and word end transpositions (*problme*). Participants first read the sentences and responded to comprehension questions while their eye movements were recorded. After the experiment participants completed a nonword circling task. Participants were presented with the same materials that they had read during the experiment, and they were asked to circle any words that they did not understand. Performance in this task, and measures of accuracy in response to comprehension questions during the main experiment, tested whether older readers are able to comprehend text including words with transposed letters. That is, these measures help reveal whether older adults are able to use flexible letter position coding.

As outlined in the introduction there were several possible outcomes. The first possibility is that despite visual declines, older adults process letter position in the same way as young adults such that the effect of transpositions on reading behavior may be equivalent for young and older adults. A second possibility is that older adults may be more dependent on fixed letter position coding, and so have difficulty reading words with transposed letters. For example, their comprehension of sentences including words with transposed letters may be poorer than for young adults, and they may circle TL nonwords in the nonword circling task, indicating that the intended meaning of the words was not understood. Alternatively, a third possibility is that older adults may be more flexible (less precise) in their letter position coding compared to young adults. If older adults do process words more holistically and rely less on the component features of a word, their eye movement behavior may be less disrupted by letter transpositions. Further, young and older adults may show differences in the degree of importance for letters in different positions compared to young adults. Letters located adjacent to spaces at the beginning and ends of words benefit from reduced visual crowding. Older readers have greater sensitivity to visual crowding (Scialfa et al., 2013) and may therefore have greater reliance on external letters. If older adults do tend to rely to a greater extent on word external letters then they may experience greater disruption than young adults when external letters are transposed, but less disruption (or even no disruption) when internal letters are transposed. Broadly, if older adults have particular difficulty with processing words with transposed letters during initial reading of words (during first-pass), then such difficulty may be linked to sublexical or lexical processes such as visual crowding, letter position coding, or lexical candidate generation. However, if older adults show particular difficulty with processing words with transposed letters only during later reading of the text (during rereading), this may be linked to postlexical processing of words, such as difficulty linked to postlexical word identification checks.

### Method

#### Participants

Twenty young adults (*M* = 19.8, *SD* = 1.6, range = 18–27 years, 14 female) and 20 older adults (*M* = 69.0, *SD* = 3.3, range = 65–76 years, 13 female) were recruited from the University of Leicester and the surrounding community. Participants were native English speakers and reported no history of reading impairment or serious eye diseases (such as advanced cataracts or macular degeneration). Participants were screened to ensure a corrected visual acuity of 20/35 or better at the viewing distance (tested using an Early Treatment Diabetic Retinopathy Study chart; Ferris & Bailey, 1996). The older adults had poorer acuity (*M* = 20/28, range = 20/20–20/35) than the young adults (*M* = 20/18, range = 20/14–20/30), *p* < .01), as is typical for these age groups (Elliott, Yang, & Whitaker, 1995). The two age groups did not differ on years of formal education (young, *M* = 15.6 years, *SD* = 1.3; older, *M* = 15.8 years, *SD* = 1.5; *p* > .05) and all reported spending several hours reading each week. Older participants were screened for normal cognitive abilities using the Montreal Cognitive Assessment (*M =* 28.1/30 *SD* = 1.1, using the standard exclusion criterion of <26/30; Nasreddine et al., 2005).

#### Materials and design

**Figure 1 fig1:**
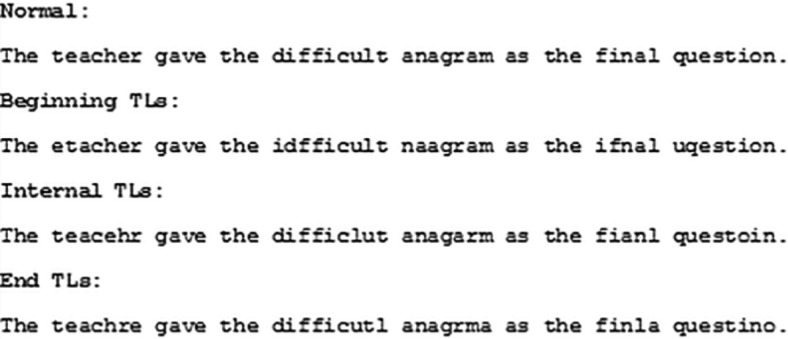
An example sentence for the normal and each of the transposed letter (TL) conditions.

Eighty sentences (adapted from White et al., 2008) were presented in four conditions, forming a 2 (age group: young, older) × 4 (text type: normal, beginning TLs, internal TLs, end TLs) mixed design (examples of each type of transposition are shown in Figure 1). Half of the sentences in the internal transposed letter condition contained internal transpositions near the beginning of the word (*porblem*) and half contained internal transpositions located near the end of the word (*probelm*; based on the internal letter transpositions employed by White et al., 2008). Sentences were presented as a single line of text and varied in length from seven to 15 words (*M* = 10.7). Transpositions were applied to all words containing five or more letters. Each sentence contained at least three words with a transposition (*M* = 4.1, range = 3–6). Eleven stimuli from the original White et al. stimuli set contained fewer than three transpositions and so were adjusted. Transpositions always involved adjacent letters. None of the transpositions resulted in the production of a real word, none retained the original spelling of the word and none were proper nouns. Transpositions did not cross morpheme boundaries. The items were counterbalanced such that participants saw an equal number of sentences from each condition. Forty percent of the sentences were followed by comprehension questions.[Fig-anchor fig1]

Following completion of the eye tracking session, participants completed a nonword circling task. Participants were instructed to circle any words that they did not understand. The stimuli for this task included the sentences that the participants had been presented with in the main experiment, with 10 additional items that included random letter string nonwords (e.g., *eoynam*). These items ensured that the task was being carried out properly, as a failure to circle these words as not understood would indicate that the task was not being performed correctly.

#### Apparatus

An SR Research EyeLink 1000 Tower Mounted Eye Tracker was used to record gaze location every millisecond. The display screen resolution was 1,024 × 768 and the refresh rate was 120Hz. Viewing was binocular, but only the right eye was tracked. At the 80 cm viewing distance, one degree of visual angle comprised approximately 3.3 characters. Black text in 15-point Courier New font was presented against a light gray background.

#### Procedure

This study received ethical approval from The University of Leicester Ethics Committee (Psychology). Before commencing the eye tracking session, participants received instructions including, “Some of the letters in some of the words may be mixed up. However, you will probably be able to guess what most of these words mean. Therefore, please concentrate on understanding the sentences to the best of your ability.” A chin and forehead rest were used to minimize head movements. Before commencing the experiment, a three-point calibration (left, center and right) and validation procedure, which covered the area of the longest sentence, was conducted. The accuracy of this calibration was checked prior to each trial, and recalibrations were performed as necessary. At the beginning of each trial a fixation cross was presented in the same position as the beginning letter of each sentence. Participants were required to fixate this cross, which triggered the presentation of the text. Participants pressed a button on a game pad to indicate that they had finished reading the sentence. For the sentences followed by a comprehension question, participants responded using the game pad to indicate a “yes” or “no” response. At the end of the session participants completed the nonword circling task, they were instructed to circle any words that they did not understand.

#### Analyses

Following standard procedures, fixations shorter than 80ms or longer than 1,200ms were discarded. This accounted for 1.9% of fixations. The data were analyzed using linear mixed effects models (LMM; Baayen, Davidson, & Bates, 2008) conducted using R (R Core Team, 2015) and the lme4 package (Bates, Mächler, & Bolker, 2011). Analyses were conducted using the normal condition as a baseline, with each transposed letter condition compared against this. These contrasts are of primary interest as they reveal the overall level of disruption caused by reading TL nonwords. These contrasts were coded using an inverse contrast matrix (such that for each contrast the transposed letter condition was coded as −.5 and the normal condition was coded as .5). In addition, the contrasts for age group were defined using sliding contrasts in the MASS package (Venables & Ripley, 2002). Both age group (young vs. older) and text type (normal vs. beginning TLs; normal vs. internal TLs; normal vs. end TLs) were included as fixed effects in the LMMs. To examine significant interactions between age group and text type, follow up contrasts were conducted which compared the normal condition and the relevant transposed letter condition separately for each age group. Importantly, the test for an interaction between age and text type (contrasts with the normal condition as the baseline) provide the strongest test of the hypotheses that older adults may be less disrupted by internal transpositions, but more disrupted by external transpositions compared to young adult readers. Furthermore, based on the findings of White et al. (2008) and Johnson and Eisler (2012),we anticipated that beginning transpositions would be the most disruptive to reading, and that end transpositions would be more disruptive than internal transpositions. To examine this, additional sliding contrasts were defined comparing the beginning to the end condition, and the end to the internal transposed letter condition. Again, sliding contrasts were also used to define age group and this produced 2 × 2 comparisons of age group and transposition type.

Log transforming the data produced the same overall pattern, and so nontransformed data are reported for greater transparency. Following current practice, a maximal random effects structure was used (Barr, Levy, Scheepers, & Tily, 2013; all models converged). Participants and stimuli were specified as crossed-random effects, age group and text type were specified as fixed factors. As sentences contained several words with transposed letters, all analyses include all words within a sentence.^1^

To minimize issues arising from multiple comparisons, discussion of the results focuses on sentence reading times (see Orquin & Holmqvist, 2018, for a discussion of the importance of carefully selecting measures), as this provides the most comprehensive measure of reading difficulty. Typically in LMM analyses, *t*/*z* values > 1.96 are interpreted as significant. However, von der Malsburg and Angele (2017) argued that the failure to adjust for multiple comparisons increases the likelihood of false positives. To adjust the critical *t*-value, the alpha threshold (0.05) was divided by the number of comparisons (10) for sentence reading times and the corresponding *t*-value was calculated (2.81). Consequently, we considered effects to be statistically significant only for *t* values that exceeded 2.81.^2^

In addition, a range of eye movement measures are also reported as these help reveal how differences in sentence reading time manifest. These measures are average fixation duration, number of fixations, number of regressive saccades (number of backward eye movements, both within and between words) number of first-pass skips (the number of words that do not receive a first-pass fixation) first-pass reading times (the sum of fixations that occurred the first time a word was encountered), and rereading times (the sum of rereading fixations).

Simulations of statistical power were conducted using the simR package in R (Green & MacLeod, 2016). The power to detect an effect of text type (normal vs. transposed) was assessed based on means and standard deviations for each type of transposition from White et al. (2008). (Note that the White et al., 2008, study included two internal transposition conditions. For these calculations the average of the two internal conditions was taken.) This confirmed that Experiment 1 had sufficient statistical power (>99%) to detect this effect and indeed that an effect of this size could also be detected with an even smaller sample size.

### Results

#### Comprehension and nonword circling task

Comprehension accuracy was high, with accuracy rates for all participants above 85% (*M =* 94%) and *t* tests revealed that accuracy did not differ by age group or by text type (all *p*s > .3). The nonword circling task revealed that participants were able to identify words well when letters were transposed. Indeed, most participants (31/40) circled no TL nonwords at all, indicating that they could understand all of the TL nonwords, and no individual participant circled more than two TL nonwords in total. The number of TL nonwords that could not be understood did not differ by age group or by the position of the transposition (*p*s > .2). In contrast, both the young and older adults circled the majority of the random letter string nonwords (e.g., *eoynam*; young adults; *M =* 9.0/10, range = 8–10/10, older adults; *M =* 9.0/10, range 8–10/10, *p* > .4). Together the sentence comprehension and nonword circling results indicate that comprehension of TL nonwords was very high for both young and older adults. These measures indicate that during the eye movement experiment words with transposed letters were likely understood by both groups.

#### Reading measures

**Figure 2 fig2:**
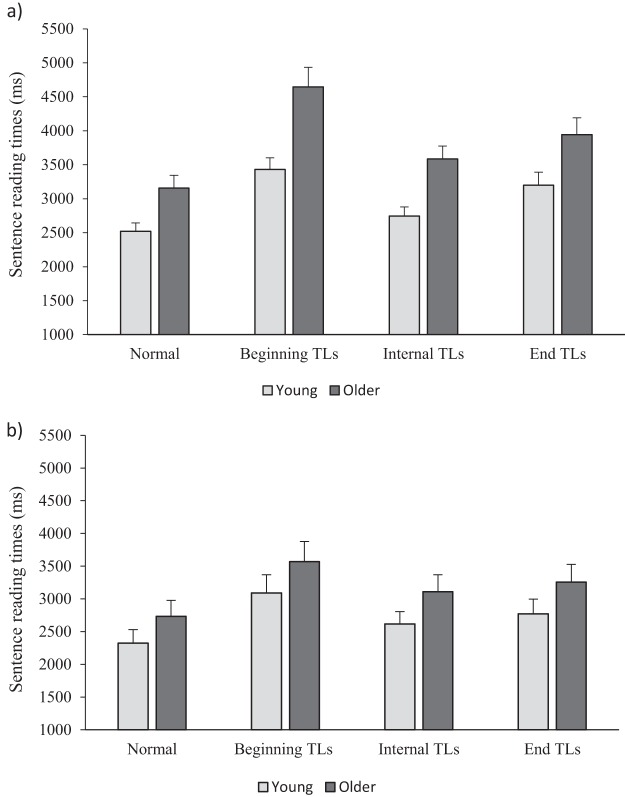
Mean sentence reading times in each condition in Experiment 1 (A) and Experiment 2 (B). Error bars represent one standard error. TL = transposed letter.

Means and standard errors are presented in Table 1. The results of the LMM for effects of age group (young vs. older) and text type (normal vs. beginning TLs; normal vs. internal TLs; normal vs. end TLs) are summarized in Table 2. Overall, older adults produced longer sentence reading times than young adults (displayed in Figure 2A). They also made more fixations, more regressive saccades and had longer rereading times than young adults. This is in line with previous evidence of age-related reading difficulties (e.g., Rayner, Reichle, et al., 2006).[Table-anchor tbl1][Table-anchor tbl2][Fig-anchor fig2]

Transpositions at beginning, internal, and end positions produced slower sentence reading times than for normally presented text. This pattern of effects was similar for the eye movement measures. There were also significant interactions between age group and text type for sentence reading time, but only for the contrast of the normal versus beginning transposed letter condition (β = 535.75, *SE* = 168.27, *t* = 3.18). Beginning transpositions resulted in greater disruption for older, compared to young, adults (see Figure 2A). This same pattern was found for number of fixations and rereading time (see Table 2).

In addition to the LMM analyses, Bayes factors (BF) were calculated to assess the strength of evidence for the interactions in Experiment 1. BF statistics are summarized in Table 3. These were computed using the BayesFactor package (Morey & Rouder, 2015) in R (R Core Team, 2015), with the scaling factor for g-priors set to 0.5 and using 100,000 Monte Carlo iterations. Participants and items were specified as random effects. Following Vandekerckhove, Matzke, and Wagenmakers (2015; adapted from Jeffreys, 1961), BFs > 3 were taken to provide weak to moderate support for a model and BFs > 10 to provide strong support, whereas BFs < 1 were taken to provide evidence against a model and in favor of the base model. The denominator model (the base model to which other models were compared) included only effects of age group and text type (normal vs. beginning TLs; normal vs. internal TLs; normal vs. end TLs) but no interaction. BFs were calculated separately for each condition compared to the normal condition. For sentence reading time, the BF analyses provided support (a high BF value) for a model with an interaction between age group and text type over a model with only main effects for the contrast of the normal text condition versus the beginning transposition condition only (normal vs. beginning TL; BF = 8216.17, normal vs. internal TL; BF = 1.03, normal vs. end TL; BF = 0.33). This pattern was also found for number of fixations and rereading time. For all other measures an additive model was preferred (i.e., a low BF value, see Table 3). This supports the main analysis demonstrating greater reading difficulty for older adults in the external beginning condition in comparison to the normal condition for measures sensitive to rereading.[Table-anchor tbl3]

To examine the influence of transpositions at different positions within a word for sentence reading times, sliding contrasts were used to produce comparisons of the beginning versus end and the end versus internal transposed letter conditions (based on previous findings: Johnson & Eisler, 2012; White et al., 2008). These contrasts revealed that greater disruption occurred for the end compared to the internal transposition condition (β = 424.80, *SE* = 80.39, *t* = 5.28) and for the beginning compared to the end transposed letter condition (β = 457.44, *SE* = 102.48, *t* = 4.46). This pattern of results is in line with previous research (e.g., Guérard et al., 2012; Johnson & Eisler, 2012; White et al., 2008), and demonstrates the importance of external letters, and beginning letters, in particular, for word recognition. Interactions between text type and age group did not reach significance for either the contrast of end versus internal transpositions (*t* = 0.51) or beginning versus end transpositions (*t* = 2.25). However, in line with the main analyses with the normal condition as a baseline, BF analyses provided support for an interaction between age group and text type for the beginning versus end contrast (BF = 10.14) but no support for an interaction for the end versus internal transposition contrast (BF = 0.09) again suggesting that older adults experienced greater disruption than young adults in the external beginning condition.

### Discussion

In line with numerous previous studies, older adults displayed longer reading times and made more regressive eye movements (Kliegl et al., 2004; McGowan et al., 2014, 2015; Paterson et al., 2013a, 2013b; Rayner, Reichle, et al., 2006; Stine-Morrow et al., 2010). Nevertheless, older adults were able to successfully comprehend words including transpositions of beginning, internal, and end letters (as demonstrated by performance in the nonword circling task and high levels of accuracy for the comprehension questions). The results of Experiment 1 therefore provide important evidence for the use of flexible letter position coding by both young and older adults. This is in line with current models of letter position coding such as SOLAR (Davis, 1999) and SERIOL (Whitney, 2001) and suggests that the basic mechanisms underlying this important aspect of word recognition are similar across the adult life span.

Importantly, in line with previous findings for young adults (Blythe et al., 2014; Johnson & Eisler, 2012; White et al., 2008), the eye movement measures show that both age groups were sensitive to transpositions in all positions within a word. Furthermore, older adults experienced greater increases in reading times for transposed letters at the beginnings and ends of words relative to those at internal positions (as the young adults did). For both young and older adults, transpositions at the beginning of words were most disruptive. These findings provide important evidence for comparable word decoding processes across adult age. The results indicate that despite changes occurring to visual abilities in advanced age, the position of letters within words remains important for word recognition. The results also provide support for the notion that external letters are particularly crucial for word identification, with the beginning letter being the most important of all (Guérard et al., 2012; Johnson & Eisler, 2012; Rayner, White, et al., 2006; White et al., 2008). End transpositions also produced more reading difficulty than internal transpositions for both young and older adults. Johnson and Eisler’s (2012) study with young adults indicated that the importance of end letters is linked to reduced lateral interference due to the space at the end of the word. Reduced lateral interference may also account for the importance of end letters for older adults, especially as studies have demonstrated the particular importance of spaces between words for older adults reading in English (McGowan et al., 2014; Rayner, Yang, Schuett, & Slattery, 2013).

Interestingly, although both age groups had most difficulty with word beginning transpositions, these caused most disruption for the older adults, as demonstrated by their much larger increase in reading times for this transposition condition. Analyses that explored this effect further by decomposing reading time into time spent initially reading words and time spent rereading words indicate that this effect may be driven by reading behavior linked to rereading. In particular, these further analyses show that differences between young and older readers were only apparent in rereading, and not during the initial (first-pass) processing of words (when letter position coding is initiated). Note, however, that the greater rereading of these words by older adults was not associated with an increase in regressive saccades by the older than younger adults.

One possibility is that older readers had more protracted difficulty identifying words with beginning letter transpositions and that this produced a larger increase in rereading that was not associated with an increase in regressive saccades to these words. Studies that have examined the rereading of words show that processes of lexical identification are triggered during both first-pass reading and rereading of words (Booth & Weger, 2013; Raney & Rayner, 1995). The interaction may therefore reflect the particular importance of beginning letter information for word recognition by older readers, either because letters in words are processed serially from left to right (e.g., Kwantes & Mewhort, 1999) or because beginning letters are important in constraining lexical candidates for words (Broerse & Zwaan, 1966; Clark & O’Regan, 1999; Hand et al., 2012; Lima & Inhoff, 1985). However, a weakness to this account is that such effects might be expected to occur during the early processing of words and so be seen in first-pass processing of words, but Experiment 1 showed no evidence of interactions with age during first-pass. A second possibility is that the particular difficulty that the older adults experience is a consequence of their greater tendency to reread words. This increased rereading appears to be typical of older adults’ reading behavior as shown previously in other studies (e.g., Rayner, Reichle, et al., 2006). Such increased rereading may be due to older adults employing more stringent postlexical checks during rereading. The disruption associated with beginning letter transpositions during rereading for older adults may therefore indicate that these more stringent postlexical checks rely especially on the word initial letters.

## Experiment 2

The aim of Experiment 2 was to provide a stronger test that first-pass letter position coding is similar across adult age groups when letter transpositions are only presented during first-pass and words are shown normally during rereading. In Experiment 1, TL nonwords were present throughout the reading process. In contrast, Experiment 2 used a gaze-contingent paradigm such that when the eyes moved past each TL nonword these words were then presented correctly (e.g., *probelm* changed to *problem*). Therefore, processing of words during rereading in Experiment 2 is likely to be based only on the correctly presented words (see Booth & Weger, 2013). If the larger effects of beginning transpositions for older adults shown in Experiment 1 relate specifically to the difficulty incurred during rereading (e.g., during postlexical checking) then such effects should be eliminated in the present experiment, as words are shown normally when reread. If the effects of age group and text type are additive in Experiment 2 then this will be consistent with the suggestion that at least initial (first-pass) letter position coding processes are not modulated by adult age.

### Method

#### Participants

Twenty young adults (*M* = 19.5 years, *SD* = 1.7, range = 18–25, 14 female) and 20 older adults (*M* = 69.2 years, *SD* = 3.5, range = 65–77, 14 female) were recruited from the University of Leicester and the surrounding community. None took part in Experiment 1. The criteria for participating were the same as in Experiment 1 and participants’ visual abilities were assessed using the same tests. Once again, older adults had poorer acuity at the viewing distance in the experiment compared to the young adults (young adults *M* = 20/19, range = 20/14–20/28; older adults *M* = 20/27, range = 20/19–20/34, *p* < .01). Participants were matched on years of education (young adults *M* = 15.3, *SD* = 1.2; older adults *M* = 15.0, *SD* = 1.4, *p* > .05), and all reported spending several hours reading each week. Older participants were screened for normal cognitive abilities using the Montreal Cognitive Assessment (*M =* 28.2/30, *SD* = 1.2 using an exclusion criterion of >26/30; Nasreddine et al., 2005). An assessment of vocabulary knowledge using the Wechsler Adult Intelligence Scale (WAIS- IV; Wechsler, 2008), showed no age group differences (young adults, *M* = 48, *SD* = 4.0; older adults, *SD* = 4.2, *M* = 49, *p* > .05, values refer to raw scores, not vocabulary size).^3^

#### Materials and design

**Figure 3 fig3:**
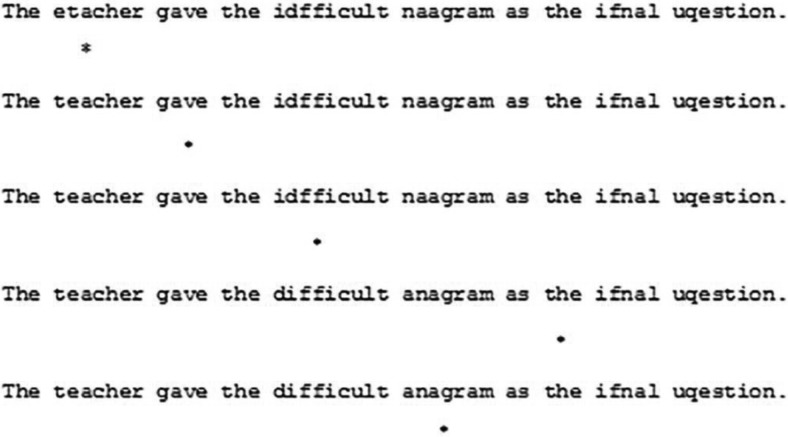
Experiment 2. A demonstration of the gaze contingent manipulation. An asterisk (*) represents the point of fixation.

The same materials as in Experiment 1 were used. Experiment 2 used a variation of the boundary gaze contingent change paradigm (McConkie & Rayner, 1975). Once a reader made a progressive eye movement beyond a TL nonword this was replaced with the correctly spelled word (see Figure 3). That is, once the eyes moved past a TL nonword, the leftward parafoveal postview of the word was always spelled correctly, and the word continued to be correctly spelled for the remainder of the trial, including during any subsequent rereading.[Fig-anchor fig3]

#### Apparatus

The apparatus was the same as that used in Experiment 1. Experiment 2 includes gaze-contingent changes, the time from when the eye moved into a region until the display change was executed was approximately 6–12 ms.

#### Procedure and analyses

The general procedure and data analyses were the same as for Experiment 1. As in Experiment 1, fixations shorter than 80 ms or longer than 1,200 ms were discarded. This accounted for 1.7% of fixations. As in Experiment 1, sentence reading time was the primary dependent measure and results for sentence reading time were considered significant only if the *t* value exceeded 2.81.

### Results

#### Comprehension

Comprehension accuracy was high, with all participants achieving an accuracy of at least 85% (*M =* 96%). As in Experiment 1, *t* tests revealed that comprehension did not differ by age group or by text type (all *p*s > .3). This further indicates that both young and older adults are able to successfully read and comprehend sentences including words with transposed adjacent letters.

#### Reading measures

Means and standard errors are presented in Table 4. The results of the LMM for effects of age group (young vs. older) and text type (normal vs. beginning TLs; normal vs. internal TLs; normal vs. end TLs) are summarized in Table 5. In Experiment 2 older adults produced longer sentence reading times than young adults. This pattern is in line with the findings of Experiment 1, as well as previous studies (e.g., Rayner, Reichle, et al., 2006).[Table-anchor tbl4][Table-anchor tbl5]

Transpositions at beginning, internal, and end positions produced slower sentence reading times than for normally presented text (displayed in Figure 2B). A similar pattern of effects was obtained for the other eye movement measures. Moreover, this pattern is in line with the findings of Experiment 1. The results therefore further demonstrate the importance of external letters and the particular importance of the beginning letter in word recognition for both young and older adults. Crucially, unlike in Experiment 1, there were no interactions between age group and any of the effects of text type.

In addition to the LMM analyses, BFs were calculated to assess the strength of evidence for the null interactions in Experiment 2. These statistics are presented in Table 6. These analyses were undertaken using the same method as in Experiment 1. In all cases, support was found for the base model over a model including an interaction between age group and text type. Thus, the interactions in Experiment 1 were eliminated in Experiment 2, young and older adults responded similarly to reading words with transposed letters for all of the measures (see Table 6).[Table-anchor tbl6]

As in Experiment 1, to examine the influence of transpositions at different positions within a word, additional models for sentence reading time were conducted with sliding contrasts that compared the beginning versus end, and the end versus internal transposed letter conditions. In line with Experiment 1, there were longer sentence reading times for the end TL condition compared to the internal TL condition (β = 153.29, *SE* = 54.12, *t* = 2.83) and for the beginning TL condition compared to the end TL condition (β = 371.12, *SE* = 63.30, *t* = 5.86). Crucially, in line with the comparisons with the normal condition as the baseline, there were no interactions with age group (*t*s < 1.4). Further, all BF analyses preferred an additive model over a model including an interaction (BFs < 0.5). Therefore, in Experiment 2, young and older adults were affected similarly by transpositions at different positions within a word.

### Discussion

In Experiment 2 older adults displayed standard adult age differences in reading, with older adults producing longer sentence reading times than young adults in line with Experiment 1 and previous research (Kliegl et al., 2004; McGowan et al., 2014, 2015; Paterson et al., 2013a, 2013b; Rayner, Reichle, et al., 2006; Stine-Morrow et al., 2010; Warrington et al., 2018).^4^ Both age groups were sensitive to transpositions in all positions within a word, as reading times were longer for each of the transposed letter conditions compared to normally presented text. However, comprehension was high and so both groups were able to successfully read the text containing words with transposed letters. Therefore, as in Experiment 1, both young and older adults displayed flexible letter position coding. In Experiment 2, all words were presented correctly once the eye moved past them and during any subsequent rereading. In line with Experiment 1, contrasts revealed that for both young and older readers beginning transpositions were more disruptive than end transpositions, and end transpositions were more disruptive than internal transpositions. These findings provide further support for the notion that external letters, particularly the beginning letters, have a privileged role in word identification (e.g., Guérard et al., 2012; Johnson & Eisler, 2012; Rayner, White, et al., 2006; White et al., 2008). Therefore word-initial letters have an important role in word recognition for both young and older adults and letter position coding processes during first-pass reading are comparable across adult age.

Crucially, in contrast to Experiment 1, age group did not interact with the position of the transposition. In Experiment 1, the transpositions remained throughout the trial, including during rereading. As the older adults generally spent more time rereading than the young adults, they were more likely to reencounter words with transposed letters that are more difficult to read. This may have disrupted their postlexical checking of words, especially in the beginning letter transposition condition. In contrast, in Experiment 2 a gaze contingent manipulation was employed such that words that included letter transpositions during first-pass reading were presented correctly once the eye moved past them. Unlike in Experiment 1, the transpositions affected the eye movement behavior of young and older adults similarly. The key finding therefore from Experiment 2, is that letter position coding during first-pass reading is equivalent for young and older adults, such that letter position coding processes are preserved in older age, at least during the initial reading of words.

## General Discussion

The findings of the present study provide clear indication that initial (i.e., first-pass) letter position coding processes are similar for both young and older adult readers. Flexible letter position coding appears to be intact in older age and both young and older adults show similar effects of letter position, with the word initial letters being the most crucial for word recognition. In Experiment 1, older adults experienced particular difficulty during rereading when word-beginning letters were transposed. The implications for older adults’ letter position coding, and possible explanations for the increased difficulty seen in rereading, are set out in detail below.

The pattern of results shown in both Experiment 1 and Experiment 2 for transpositions at different positions within words are in line with previous studies examining these processes for young adults. The finding that older adults can comprehend words with transposed letters and that they can do this with relatively little disruption to the reading process, indicates that they have flexible letter position coding. These findings are in line with the predictions of word encoding models that employ flexible letter position coding (e.g., SERIOL, Whitney, 2001; SOLAR, Davis & Bowers, 2006; The Overlap Model, Gómez et al., 2008). Furthermore, transpositions of letters in both internal and external positions disrupted reading, indicating that the position of both internal and external letters is important for both young and older adults. This finding is particularly important as it reveals that despite a range of visual declines in advanced age, such as greater sensitivity to effects of crowding (Scialfa et al., 2013), the position of internal letters remains important for normal word recognition for older adults. This is also in spite of evidence that word recognition in older age may be more holistic and automatized (Lien et al., 2006; Spieler & Balota, 2000).

Importantly, the results also show that effects of letter position, previously shown for young adults, also hold for older adults. Older adults show a greater increase in reading times when letters at the beginning or the end of a word are transposed relative to when internal letters are transposed. This pattern is identical to the pattern typically observed for young adults. The findings support the suggestion that external letters are more important than internal letters, and word beginning letters are especially important (Aschenbrenner et al., 2017; Carr et al., 1976; Forster, 1976; Guérard et al., 2012; Johnson & Eisler, 2012; Jordan et al., 2003; Rayner & Kaiser, 1975; Rayner, White, et al., 2006; White et al., 2008). Crucially, these results extend previous findings with young adults, demonstrating that this pattern also holds for older adults. Johnson and Eisler’s (2012) study indicated that for young adults the importance of word ending letters may be due to the following space reducing effects of lateral interference between letters. Similar factors may well account for the importance of word ending letters for older adults, especially given older adults’ sensitivity to crowding (Scialfa et al., 2013). In addition to perceptual factors such as reduced lateral interference, word initial letters are likely to be especially important for word recognition. For example, similar to young adults, word initial letters may be important due to constraining possible lexical candidates (e.g., Clark & O’Regan, 1999), serial scanning of letters within words (e.g., Kwantes & Mewhort, 1999) or because word initial letters may have an earlier (Whitney, 2001) or a privileged role (Gómez et al., 2008) in letter position encoding (see Aschenbrenner et al., 2017; Johnson & Eisler, 2012).

Overall, the results of the experiments presented here indicate that letter position coding in older adults is operating in a similar way to young adults, with a similar pattern of importance for beginning, internal, and end letters within a word. That is, letter position coding within words appears to remain relatively intact in older age for both word internal and word external letters. Therefore, current model-based explanations of letter position coding (e.g., SERIOL, Whitney, 2001; SOLAR, Davis & Bowers, 2006; The Overlap Model, Gómez et al., 2008) may be applicable to both young and older adults as mechanisms underlying letter position coding appear to be similar for both age groups.

However, although letter position coding generally appears to be intact in older age, in Experiment 1 reading difficulty incurred due to the word-beginning transpositions was especially pronounced during rereading for the older adults. It seems likely that there are two components to this effect. The first is associated with a cost due to the reinitiation of lexical identification processes during the rereading of words (see Booth & Weger, 2013; Raney & Rayner, 1995). Consequently, older adults may incur a larger cost during reading compared to young adults simply because they reread more, such that the challenge of letter position coding arise again when reading the words for a second time (a “double whammy” effect).^5^ In addition, a further key component may be more stringent postlexical checking of words during rereading by older adults, which may especially rely on the accuracy of the word beginning letters. The interaction effect observed in Experiment 1 may have arisen because beginning letter transpositions especially interfered with the more stringent postlexical checks undertaken by older readers.

In sum, the present study provides novel insight into letter position coding across adulthood. The overall pattern of results suggests that during normal reading young and older adults are likely to process letter position similarly, especially during first-pass reading. The results further highlight that word initial letters play a particularly important role in word recognition for both young and older adults.

## Figures and Tables

**Table 1 tbl1:** Experiment 1: Means (SEs) for the Primary Measure of Sentence Reading Time and the Additional Eye Movement Measures

Measure	Normal	Beginning TLs	Internal TLs	End TLs
Sentence reading time (ms)				
Young	2,521 (122)	3,431 (172)	2,744 (133)	3,200 (191)
Older	3,156 (188)	4,647 (285)	3,585 (189)	3,983 (248)
Average fixation duration (ms)				
Young	238 (5)	255 (6)	246 (5)	247 (5)
Older	238 (6)	260 (6)	247 (6)	249 (6)
Number of fixations				
Young	10.3 (.4)	13.0 (.6)	10.8 (.4)	12.5 (.6)
Older	12.1 (.6)	16.2 (.8)	13.2 (.6)	14.6 (.8)
Number of regressive saccades				
Young	2.3 (.2)	3.0 (.3)	2.3 (.2)	2.7 (.2)
Older	3.9 (.3)	4.7 (.4)	3.9 (.3)	4.6 (.4)
Number of first-pass skips				
Young	3.4 (.2)	3.1 (.2)	3.4 (.2)	3.1 (.2)
Older	4.0 (.3)	3.7 (.2)	3.8 (.3)	3.7 (.3)
First-pass reading time (ms)				
Young	2,002 (87)	2,432 (120)	2,124 (95)	2,346 (112)
Older	1,918 (79)	2,408 (115)	2,120 (88)	2,309 (104)
Rereading time (ms)				
Young	508 (71)	994 (81)	579 (59)	838 (107)
Older	1,081 (102)	1,932 (186)	1,254 (122)	1,430 (167)
*Note*. TLs = transposed letters.				

**Table 2 tbl2:** Experiment 1: Linear Mixed Effects Model Statistics

Measure	Sentence reading time (ms)	Average fixation duration (ms)	Number of fixations	Number of regressive saccades	Number of first-pass skips	First-pass reading time (ms)	Rereading time (ms)
Intercept							
β	3,400.84	246.42	12.85	3.38	3.55	2,186.86	1,009.32
*SE*	141.11	4.02	.45	.20	.17	73.06	82.77
*t*	24.10^a^	61.37	28.71	16.48	21.10	29.93	13.04
Age group							
Young vs. older							
β	839.95	.40	2.39	1.70	.53	78.53	610.28
*SE*	269.18	7.89	.85	.40	.27	141.83	155.55
*t*	3.12^a^	.05	2.83	4.21	1.94	.55	3.92
Text type							
Normal vs. beginning TLs							
β	1,207.56	19.56	3.39	.78	.29	443.91	624.55
*SE*	105.90	2.00	.30	.17	.06	43.77	89.70
*t*	11.40^a^	9.77	11.07	4.62	4.53	10.14	6.96
Normal vs. internal TLs							
β	325.32	8.54	.78	.02	.10	162.44	127.73
*SE*	58.03	1.80	.19	.11	.05	25.19	56.34
*t*	5.61^a^	4.75	4.00	.15	1.82	6.45	2.27
Normal vs. end TLs							
β	750.12	9.83	2.31	.48	.33	357.83	329.57
*SE*	94.85	1.69	.30	.12	.05	33.54	73.41
*t*	7.91^a^	5.80	7.75	3.85	6.71	10.67	3.95
Age Group × Text Type							
Age Group × Normal vs. Beginning TLs							
β	535.75	5.37	1.36	.17	.04	22.37	442.16
*SE*	168.27	3.90	.47	.31	.12	82.21	153.06
*t*	3.18^a^	1.38	2.86	.55	.34	.27	2.89
Age Group × Normal vs. Internal TLs							
β	207.69	1.50	.68	.08	.19	83.82	108.40
*SE*	105.31	3.53	.34	.21	.10	50.07	108.30
*t*	1.87	.43	1.88	.38	1.73	1.67	1.00
Age Group × Normal vs. End TLs							
β	127.43	2.48	.32	.15	.06	28.57	12.89
*SE*	168.82	3.34	.52	.23	.10	65.35	157.90
*t*	.76	.74	.62	.65	.63	.44	.08
*Note.* TLs = transposed letters. Additional eye movement measures are included for completeness (see Footnote 2). Random effect variance for sentence reading times: participants-variance = 711,827, *SD* = 844; items-variance = 144,085, *SD* = 380.							
^a^ Significant effects (*t* value > 2.81) in sentence reading times.							

**Table 3 tbl3:** Bayes Factor Values (BF) for Experiment 1

Measure	Normal vs. beginning TLs	Normal vs. internal TLs	Normal vs. end TLs
Sentence reading time (ms)	8,216.17	1.03	.33
Average fixation duration (ms)	.49	.08	.14
Number of fixations	89.83	.90	.16
Number of regressive saccades	.11	.09	.11
Number of first-pass skips	.08	.61	.11
First-pass reading time (ms)	.17	.89	.19
Rereading time (ms)	302.73	.31	.10
*Note.* TLs = transposed letters. Values refer to the strength of evidence in favor of a model including an interaction over an additive model. BFs > 3 provide weak to moderate support for a model and BFs > 10 to provide strong support, whereas BFs < 1 provide evidence against a model and in favour of the base (i.e. additive) model.			

**Table 4 tbl4:** Experiment 2: Means (SEs) for the Primary Measure of Sentence Reading Time and the Additional Eye Movement Measures

Measure	Normal	Beginning TLs	Internal TLs	End TLs
Sentence reading time (ms)				
Young	2,324 (223)	3,091 (309)	2,617 (256)	2,772 (267)
Older	2,735 (203)	3,568 (269)	3,111 (176)	3,258 (219)
Average fixation duration (ms)				
Young	213 (8)	234 (9)	222 (9)	224 (9)
Older	226 (7)	247 (7)	235 (7)	239 (7)
Number of fixations				
Young	9.3 (.6)	11.3 (.9)	10.1 (.6)	10.6 (.8)
Older	10.4 (.6)	12.6 (.9)	11.5 (.6)	11.8 (.6)
Number of regressive saccades				
Young	2.4 (.3)	2.9 (.4)	2.5 (.4)	2.6 (.5)
Older	2.8 (.4)	3.4 (.5)	3.1 (.5)	3.1 (.4)
Number of first-pass skips				
Young	4.6 (.2)	4.6 (.2)	4.5 (.2)	4.5 (.2)
Older	4.9 (.2)	4.7 (.2)	4.7 (.2)	4.5 (.2)
First-pass reading time (ms)				
Young	1,795 (150)	2,213 (174)	1,976 (158)	2,075 (170)
Older	1,933 (82)	2,373 (115)	2,143 (91)	2,225 (100)
Rereading time (ms)				
Young	467 (89)	772 (128)	587 (121)	629 (116)
Older	670 (105)	939 (134)	707 (79)	770 (110)
*Note.* TLs = transposed letters.				

**Table 5 tbl5:** Experiment 2: Linear Mixed Effects Model Statistics

Variable	Sentence reading time (ms)	Average fixation duration (ms)	Number of fixations	Number of regressive saccades	Number of first-pass skips	First-pass reading time (ms)	Rereading time (ms)
Intercept							
β	2953.88	229.91	11.01	2.65	4.64	2096.08	674.13
*SE*	178.91	5.36	.53	.20	.17	97.88	77.74
*t*	16.51^a^	42.90	20.59	13.12	27.47	21.41	8.67
Age group							
Young vs. older							
β	495.79	13.72	1.33	.80	.08	177.03	112.27
*SE*	350.00	10.62	1.04	.40	.27	191.65	153.19
*t*	2.82^a^	1.30	1.29	2.50	.30	.92	2.73
Text type							
Normal vs. beginning TLs							
β	859.69	20.65	2.23	.69	.16	454.40	326.54
*SE*	81.53	2.04	.22	.12	.06	33.84	54.10
*t*	10.54^a^	10.11	10.04	5.92	2.89	13.43	6.04
Normal vs. internal TLs							
β	335.00	8.60	.90	.20	.07	198.68	117.37
*SE*	52.46	1.62	.17	.10	.05	24.94	61.67
*t*	6.39^a^	5.31	5.19	1.97	1.56	7.97	1.90
Normal vs. end TLs							
β	488.55	11.71	1.36	.21	.26	311.45	149.45
*SE*	52.16	1.85	.16	.09	.05	31.36	47.71
*t*	9.37^a^	6.34	8.52	2.28	4.81	9.93	3.13
Age Group × Text Type							
Age Group × Normal vs. Beginning TLs							
β	235.28	2.43	.60	.31	.08	87.02	84.11
*SE*	129.86	3.30	.34	.20	.10	58.28	100.57
*t*	1.39	.73	1.67	1.53	.91	1.45	.84
Age Group × Normal vs. Internal TLs							
β	189.08	2.14	.68	.28	.09	71.68	4.62
*SE*	104.03	2.98	.39	.20	.10	49.32	120.56
*t*	1.61	.72	1.37	1.42	.91	1.45	.04
Age group × Normal vs. End TLs							
β	107.34	2.48	.20	.14	.17	52.14	22.04
*SE*	98.07	3.48	.31	.19	.10	59.34	92.33
*t*	1.09	.71	.63	.73	1.67	.88	.23
*Note.* TLs = transposed letters. Additional eye movement measures are included for completeness (see Footnote 2). Random effect variance for sentence reading times: participants-variance = 1,215,008, *SD* = 1102. items-variance = 110,501, *SD* = 332.4.							
^a^ Significant effects (*t* value > 2.81) in sentence reading times.							

**Table 6 tbl6:** Bayes Factor Values (BF) for Experiment 2

Measure	Normal vs. beginning TLs	Normal vs. internal TLs	Normal vs. end TLs
Sentence reading time (ms)	.80	.99	.19
Average fixation duration (ms)	.07	.11	.09
Number of fixations	.31	.76	.05
Number of regressive saccades	.12	.10	.12
Number of first-pass skips	.04	.05	.21
First-pass reading time (ms)	.38	.21	.09
Rereading time (ms)	.18	.49	.10
*Note.* TLs = transposed letters. Values refer to the strength of evidence in favor of a model including an interaction over an additive model. BFs > 3 provide weak to moderate support for a model and BFs > 10 to provide strong support, whereas BFs < 1 provide evidence against a model and in favor of the base (i.e. additive) model.			

## Footnotes

1 Note that each trial included multiple words with transposed letters. Therefore, the interpretation of the time course of effects for individual words is limited, as any effects (e.g., in first fixation duration) could be due to transpositions within previously read words within a sentence. Nevertheless, analyses were undertaken for a critical word within each item (this word was always at least five letters long, and so always included a transposition) and also for all words within each item including a transposition. In both sets of analyses, for both Experiments 1 and 2, the pattern of results was consistent with the sentence-level analyses.

2 Note that there are alternative methods for performing these adjustments, such as correcting for the number of eye movement measures computed. However, because of the number of contrasts required here, correction for the number of tests for our primary variable of interest, sentence reading time, was deemed most appropriate. Note that if correction were to be applied for the number of eye movement measures, the corrected *t* value would be 2.70 (hence the pattern of results would be identical).

3 Age-adjusted scores also show no significant age differences in vocabulary between the two groups (young adults *M* = 15.1, older adults *M* = 14.1, *p* > .05).

4 Whereas older adults displayed a pattern of eye movement behavior generally consistent with previous studies (e.g., Kliegl et al., 2004), previous research has reported greater word skipping for older adults (e.g., McGowan et al., 2014). For the studies reported here there were no significant effects of age on word skipping. There were, however, numerical patterns in the same direction as previously reported.

5 The possible effect of repeated resampling of words resulting in inflated effects of manipulations for measures of rereading may be important for a variety of experimental manipulations. Future research should aim to establish the prevalence of possible “double whammy” effects in experimental findings for older adults, and with other groups of readers who show higher rates of regressions.
